# When Effective Therapies Collide: Aorto-Esophageal Fistula After Thoracic Radiotherapy and Anti-VEGF TKI in a Long-Survivor with mRCC

**DOI:** 10.15586/jkc.v13i2.448

**Published:** 2026-04-23

**Authors:** Simone Rota, Valentina Guadalupi, Camilla Damonte, Tommaso Cascella, Edoardo Borsotti, Giuseppe Procopio

**Affiliations:** 1 Genitourinary Medical Oncology, Fondazione IRCCS Istituto Nazionale dei Tumori, Milan, Italy;; 2 Department of Interventional Radiology, Fondazione IRCCS Istituto Nazionale dei Tumori, Italy;; 3 Gastroenterology and Digestive Endoscopy Unit, Fondazione IRCCS Istituto Nazionale dei Tumori, Via Venezian 1, Milan, Italy

**Keywords:** aorto-esophageal fistula, long survivors, renal cell carcinoma, stereotactic body radiotherapy, tyrosine kinase inhibitors

## Abstract

Advances in systemic therapies have improved survival in metastatic renal cell carcinoma (mRCC), leading to a growing population of long-term survivors who may receive both radiotherapy (RT) and tyrosine kinase inhibitors (TKIs) during their disease course. Both treatments induce vascular and mucosal toxicity, and their biological effects may overlap, increasing the risk of rare but life-threatening complications such as aorto-esophageal fistula (AEF). Herein, we present the case of a 47-year-old man with mRCC treated with nephrectomy and repeated pulmonary metastasectomies, followed by sunitinib, mediastinal stereotactic body radiotherapy (SBRT), and later cabozantinib for hepatic progression. Five years after thoracic RT and shortly after initiating cabozantinib, the patient developed massive hematemesis due to an AEF. Management included thoracic endovascular aortic repair (TEVAR), esophageal stenting, and prolonged antimicrobial therapy. Despite initial stabilization, recurrent fistulization and infections led to progressive deterioration and death 7 months later. This case underscores the catastrophic potential of RT–TKI interaction in long-term survivor patients. Sequential exposure can transform subclinical vascular injury into fatal outcomes. Risk stratification, nonconcurrent scheduling of RT and anti-VEGF therapy, and vigilant long-term monitoring are essential. Integration of multidisciplinary and palliative approaches is necessary to balance treatment efficacy with safety.

## Introduction

Over the past decade, survival outcomes for metastatic renal cell carcinoma (mRCC) have improved substantially, driven by advances in systemic therapy, particularly immune checkpoint inhibitors (ICIs) and ICIs + tyrosine-kinase inhibitors (TKIs) combinations. Indeed, in real-world cohorts of actively treated metastatic clear cell RCC (mccRCC), median overall survival increased from 25 to 41 months when comparing 2010–2016 with 2017–2020 (1). Moreover, across real-world population-based analyses that also include untreated patients and non-clear cell histology, median overall survival improved from 8 to 11 months with the transition from the pre-ICI to the ICI era, with a small proportion of long-term survivors, who can experience durable disease control on therapy without progression. However, antiangiogenic TKIs, such as lenvatinib and cabozantinib, remain the backbone of the mccRCC therapeutic landscape. It is well known that their mechanism of action in this histology translates into high efficacy but can also compromise the vascular integrity of normal tissue and wound healing. While most toxicities are reversible or manageable, severe vascular and gastrointestinal complications, including hemorrhage, perforation, and fistula formation, have been reported in up to 1% of treated patients (1–6).

On the other hand, radiotherapy (RT) has a well-known and fundamental role in the management of mccRCC, particularly for patients with oligometastatic or oligoprogressive disease. Stereotactic body radiotherapy (SBRT) achieves excellent local control and durable palliation, although late toxicities may occur. Indeed, high-dose hypofractionated irradiation can induce progressive vascular injury, chronic ischemia, and fibrosis of surrounding tissues. In central thoracic sites, such as the mediastinum or esophagus, these processes may culminate in structural fragility that persists long after apparent recovery. Late radiation-induced esophageal injury can manifest months to years after exposure, broadening the spectrum from mucosal ulceration to catastrophic aorto-esophageal fistula (AEF) formation (7, 8).

When patients receive a systemic line of therapy with TKI and RT during their oncological pathway, the biological collateral effect of these approaches may converge, with an increased risk in long-term survivors. Each modality alone carries distinct vascular and mucosal toxicities (7–9); when combined over time, they can act synergistically to amplify tissue vulnerability. The 2025 ESMO–ESTRO collaborative framework underlined the need for site-specific risk assessment and careful temporal coordination when integrating RT with targeted or immune-based agents, particularly in cavity-containing organs such as the esophagus, airways, and gastrointestinal tract (10). The present case illustrates this paradigm, an AEF arising in a long-term survivor with mRCC who had previously undergone mediastinal SBRT and was subsequently treated with cabozantinib. This clinical scenario underscores the necessity of proactive, multidisciplinary vigilance to anticipate and manage interaction-driven late complications.

## Case Presentation

A 47-year-old man with no significant comorbidities presented with a left renal mass radiologically suspicious for RCC and underwent left radical nephrectomy in January 2015. Histopathological examination was consistent with Grade 2 clear cell renal cell carcinoma (ccRCC) (pT3aN0M0, TNM 7th).

During surveillance, in September 2016, an 8 mm–sized pulmonary nodule was detected in the left upper lobe, leading to a metastasectomy that confirmed mccRCC with R0 resection.

In October 2017, a new pulmonary metastasis was removed by VATS wedge resection (R0). In December 2017, the patient was enrolled in an experimental protocol investigating adjuvant therapy with atezolizumab versus placebo. He received five treatment cycles between January and April 2018.

In April 2018, a restaging CT scan revealed disease progression, with an enlarging mass at the left pulmonary hilum and lymphadenopathy in the aortopulmonary window. This led to withdrawal from the clinical trial and initiation of first-line systemic therapy with sunitinib.

Subsequent imaging showed stable disease, followed by a marked reduction in thoracic lymphadenopathy, which was maintained until early 2019. In June 2019, in light of this favorable response, a treatment break from sunitinib was agreed upon.

Because of residual lymphadenopathy, SBRT with CyberKnife was performed in July 2019, targeting the paratracheal and hilar regions (60 Gy in 8 fractions). Follow-up imaging in September 2019 showed a decrease in lymph node size, consistent with a favorable treatment response.

In August 2022, routine surveillance CT revealed a 32 mm pancreatic lesion in the body–tail region, associated with dilation of the main pancreatic duct. Endoscopic ultrasound-guided biopsy confirmed mccRCC. Sunitinib therapy was therefore restarted in September 2022, achieving a partial response.

Between February and March 2023, the patient underwent stereotactic RT targeting the pancreatic lesion (44.25 Gy in 15 fractions). Initial post-RT imaging showed stability; however, in June 2024, CT revealed disease progression with approximately 20 hepatic metastases, the largest measuring 3 cm. From January to May 2024, the patient received nivolumab 480 mg (8 cycles), but due to subsequent hepatic progressive disease, third-line therapy with cabozantinib was started, resulting in a reduction in both the size and density of hepatic lesions by October 2024.

**Figure 1:**
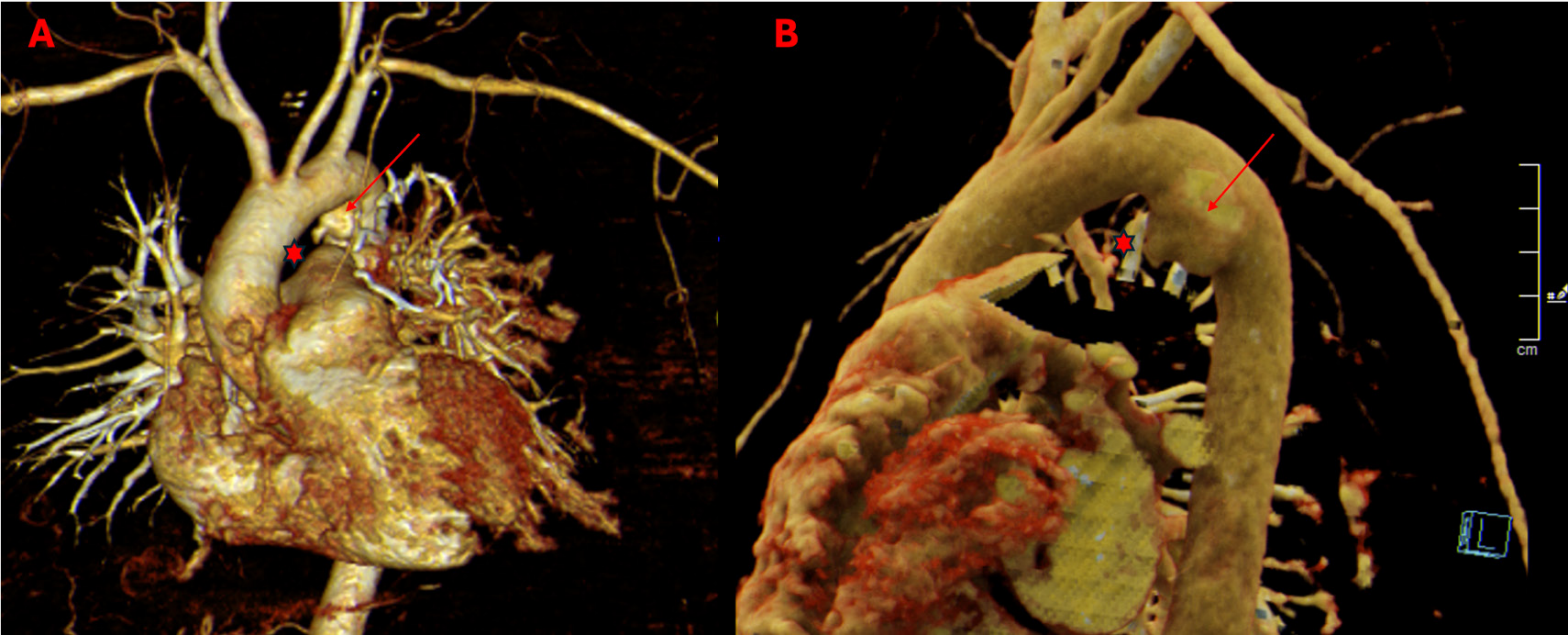
(A–B) 3D reconstruction of the AEF. A marked dilation of the aortic arch is evident, clearly showing the site of fistulous tract formation. The red star indicates the position of the esophagus in relation to the aortic fistula. For reconstructive and visualization reasons, a 3D reconstruction of the esophagus is not shown in the figure.

**Figure 2:**
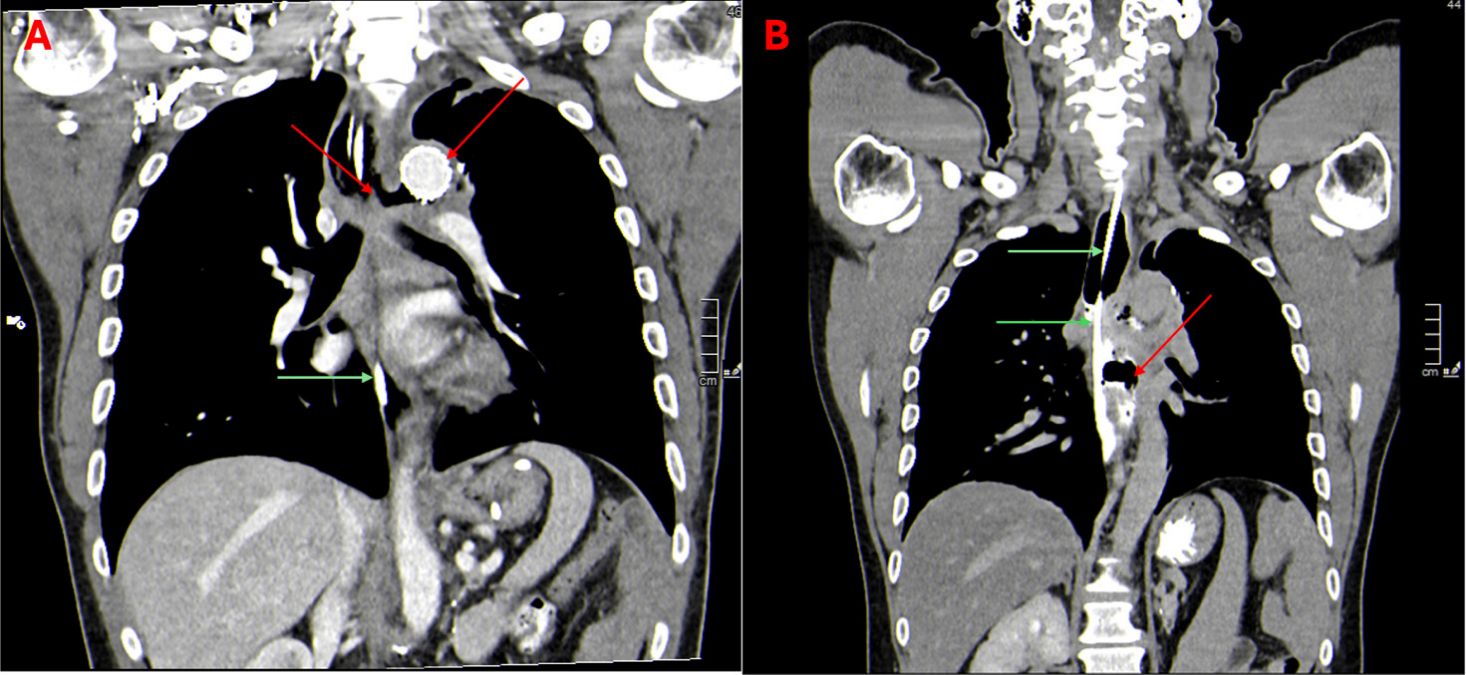
(A) Aortic prothesis inserted during the acute phase of the complication (red arrow). Residual aorto-esophageal fistula is also visible, and a nasogastric tube can be seen at the esophageal level (green arrows). (B) Evidence of an esophago-mediastinal fistulous tract (red arrow). A large accumulation of iodinated contrast medium is visible within the cavity.

**Figure 3:**
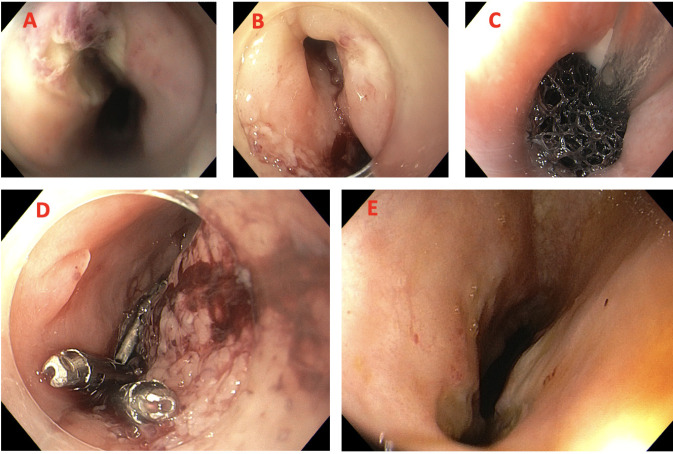
(A) Esophageal fistula with evidence of fibrin and traces of blood; (B) Esophageal fistula after the removal of esophageal stent; (C) Endoluminal vacuum therapy (Suprasorb CNP endo); (D) Endoscopic clipping of the orifice - granulation tissue following VAC therapy; E: Persistent fistula and clips dislodgement.

On November 11, 2024, the patient presented with hematemesis and hemorrhagic shock due to the development of a postradiation AEF, requiring ICU stabilization and endovascular aortic stent-graft implantation (Figure 1). Esophageal surgical repair was excluded, as a surgical approach was deemed infeasible. Management involved endoscopic monitoring of the esophageal fistula and broad-spectrum antibiotic and antifungal therapy for mediastinitis. Nasoesophageal and nasoduodenal tubes were used for aspiration and enteral nutrition (Figure 2). The first endoscopic approach to the esophageal fistula was the placement of a fully covered, antimigration Niti-S Beta 2 esophageal stent (20 × 80 mm) (Figures 3A and 3B).

During hospitalization, the patient developed bibasilar pneumonia, likely secondary to aspiration during hematemesis. The antibiotic regimen was adjusted, switching from piperacillin–tazobactam to meropenem. Radiological follow-up showed correct positioning of the esophageal stent, with no evidence of contrast leakage. In the following days, the patient’s clinical condition improved, thanks to the medical therapy and physical rehabilitation, although basal lung mobility remained limited. After refusing jejunostomy placement following surgical consultation, the patient was discharged on December 11, 2024. Initially, the quality of life was acceptable, with inflammatory markers showing a progressive decline.

On January 15, 2025, the patient presented to the Emergency Department with oppressive chest pain caused by the recurrence of mediastinitis, as confirmed by CT, which also demonstrated an early leak through the aortic stent-graft, accounting for device failure and the clinical presentation. The pain was oppressive, interscapular, radiated anteriorly, and poorly responsive to opioid analgesia. Broad-spectrum antibiotics (piperacillin–tazobactam and daptomycin) and analgesic therapy were started.

Following endoscopic reassessment, considering the failure of stenting and the need for drainage of the periprosthetic abscess, the esophageal stent was safely removed. The esophageal defect was subsequently managed with endoluminal negative pressure vacuum therapy (Suprasorb CNP endo) (Figure 3C). After several device replacements, progressive reduction and debridement of the cavity were observed; however, the fistula persisted. Considering the patient’s poor tolerance to repeated endoscopic procedures, endoscopic clipping with Mantis clips was attempted without success (Figures 3D and 3E).

Because of the incomplete resolution of the fistula, a gastrojejunostomy was performed under fluoroscopic guidance on February 27, 2025.

On March 03, 2025, the patient was discharged with ongoing long-term antibiotic therapy. Restaging CT performed in the following month showed stability of the oncological disease.

On May 26, 2025, the patient was once again hospitalized due to severe anemia caused by bleeding from the gastrojejunostomy site. CT showed a paraesophageal fluid collection and bilateral pulmonary consolidations. Initial management included multiple blood transfusions and antibiotic (piperacillin–tazobactam and daptomycin) and antifungal (caspofungin) therapies. During hospitalization, the patient also developed hematemesis with hemorrhagic shock, managed with blood transfusions, vasopressors, colloid infusions, and oxygen therapy. Surgery was deemed not feasible because of an unfavorable risk–benefit ratio. Therefore, on May 29, 2025, a new esophageal stent was placed.

In the following days, the patient’s clinical condition progressively deteriorated, with new episodes of hematemesis causing severe anemia and worsened pulmonary infection with fever and dyspnea at rest. Owing to the patient’s unresponsiveness to maximal medical therapy, palliative sedation was initiated. The patient passed away on June 14, 2025.

## Discussion

The case presented highlights the possibility of serious and life-threatening late complications arising from the interaction between RT and anti-VEGF TKI in a long-term survivor with mRCC. Although both modalities can independently improve survival, their overlapping biological effects can converge on vulnerable tissues, potentially transforming subclinical damage into fatal outcomes, even years after the treatment itself.

The pathophysiology of AEF following RT has already been described in the literature. Indeed, late radiation injury leads to endarteritis obliterans, progressive ischemia, and fibrotic remodeling of the esophageal and aortic walls. Over time, these chronic injuries, together with overlapping inflammatory or mechanical insults, can lead to erosion and fistulization (7, 8). Reported latency periods extend from months to several decades, with cases documented up to 19 years post-radiation (11).

On the other hand, anti-VEFG TKIs are responsible for another type of tissue damage. In fact, by blocking VEGF-mediated capillary repair, these agents delay healing and reduce mucosal resilience at the same time. Intriguingly, when administered to patients with preexisting radiation damage, the risk appears multiplicative rather than additive.

Evidence in mRCC is extremely limited and mainly restricted to isolated case reports. Among these, Basille et al. described a bronchial fistula occurring after mediastinal RT followed by sunitinib, while other heterogeneous fistula or perforation events have also been reported during antiangiogenic TKI therapy in RCC (12, 13). Because RCC-specific evidence remains so scarce, reports from other malignancies may help to better contextualize this complication. In thyroid cancer, aerodigestive fistulas have been described during antiangiogenic TKI treatment, particularly in previously irradiated patients or in the presence of local tissue disruption (10). Similarly, tracheoesophageal fistula has been reported during cabozantinib therapy in hepatocellular carcinoma (4). Taken together, these observations support the hypothesis that prior radiation-related vascular and mucosal injury may create a vulnerable substrate on which anti-VEGF therapy further impairs tissue repair and promotes fistulization.

The present case mirrors these patterns, with a long latency from RT (5 years) and a short exposure to cabozantinib preceding ulceration and fistulization.

The 2025 ESMO–ESTRO consensus framework offers a structured approach to the evaluation of RT–TKI combinations. It recommends a risk-stratified algorithm based on anatomical site, proposing nonconcurrent administration or “washout” intervals in cavity organs such as the esophagus, trachea, or bowel. When feasible, anti-VEGF therapy should be deferred or paused for several weeks before and after thoracic RT. At the same time, patients with prior RT should be counseled on the risks of delayed mucosal injury when initiating TKI therapy and monitored closely for early symptoms. However, as demonstrated by the case described here, even taking all these aspects into account may not be sufficient to prevent serious complications. Among these, management of AEF remains challenging.

Thoracic endovascular aortic repair (TEVAR) is the cornerstone of emergency control, providing immediate hemostasis and reducing the risk of infection and recurrence. However, persistent contamination of the prosthesis may lead to septic complications and rebleeding. Several series advocate a staged approach: TEVAR as the bridge to subsequent esophageal repair, whether surgical or endoscopic (11, 14). The described patient’s course, involving TEVAR followed by covered stenting, negative-pressure therapy, and clip closure, reflects this strategy and aligns with previous published data. Furthermore, long-term antimicrobial suppression remains justified when definitive eradication is unfeasible, particularly with infected endografts in situ. Traditional adverse event frameworks often consider toxicities as isolated phenomena, but this case demonstrates the importance of interaction-based thinking. Patients with prior high-dose thoracic RT constitute a distinct risk group when exposed to VEGF inhibition. Documenting RT dose–volume parameters, mapping irradiated fields, and integrating this information into systemic treatment planning is fundamental.

Multidisciplinary collaboration among oncologists, radiotherapists, vascular and gastroenterological teams is essential to anticipate these scenarios, wherever possible.

Finally, this case also raises ethical and palliative considerations. Once the life-threatening event has been controlled, decisions regarding further interventions must balance potential benefit against quality of life, infection risk, and overall prognosis. Early integration of palliative care in the trajectory facilitates shared decision-making, as occurred in the case presented herein during the later stages.

## Conclusions

In this long-term survivor with mRCC, the development of a catastrophic AEF after sequential exposure to thoracic RT and anti-VEGF TKI therapy strongly suggests a clinically relevant interaction between prior radiation-related tissue vulnerability and subsequent VEGF inhibition. The event underscores the importance of treatment interactions in an era where patients survive long enough to accumulate multiple modalities. Preventive strategies should include nonconcurrent scheduling of RT and antiangiogenic agents in high-risk anatomical sites, meticulous documentation of prior RT fields, and active surveillance for early symptoms of esophageal injury. Finally, awareness of this interaction should encourage the integration of survivorship and palliative perspectives into long-term mRCC care.

## Outside the submitted work

Giuseppe Procopio reported consulting or advisory role—Accord, Amgen, Astellas, AstraZeneca, Bayer, Bristol-Myers Squibb, Eisai, EUSA Pharma, Ipsen, Janssen, Lilly, Merck Sharp & Dohme, MSD Oncology, Novartis, Pfizer, Roche Genentech; Research funding—Astellas Pharma, Gilead, Ipsen, Janssen Oncology, MSD; Travel, accommodations, expenses—AAA/Novartis, Ipsen, Janssen, Recordati.

No potential conflicts of interest were disclosed by other authors.

## Consent Statement

Since no identifiable information is presented and the patient died in 2025, informed consent could not be obtained. According to the Italian law and institutional policies for a tertiary cancer center dedicated to oncologic care and research, the use of fully anonymized clinical data for research purposes is considered implicitly authorized.
